# Ecological risk and source identifications of heavy metals contamination in the water and surface sediments from anthropogenic impacts of urban river, Indonesia

**DOI:** 10.1016/j.heliyon.2023.e15485

**Published:** 2023-04-14

**Authors:** Lintang Nur Fadlillah, Sri Utami, Alfina Ayu Rachmawati, Galih Dwi Jayanto, M. Widyastuti

**Affiliations:** a Laboratory of Hydrology and Environmental Climatology, Department of Environmental Geography, Faculty of Geography, Universitas Gadjah Mada, Yogyakarta, Indonesia; b Department of Environmental Geography, Faculty of Geography, Universitas Gadjah Mada, Yogyakarta, Indonesia; c Master Program on Planning and Management of Coastal Area and Watershed, Faculty of Geography, Universitas Gadjah Mada, Yogyakarta, Indonesia

**Keywords:** Ecological risk, Environmental risk, River pollution, Sediment quality, Toxic metals

## Abstract

Heavy metal pollution in urban rivers corresponds to anthropogenic impacts. Considering the environmental importance of the Winongo River for domestic use, agriculture, and fisheries, a comprehensive study of heavy metal contamination in this river needs to be conducted. This research focused on the assessment of heavy metal in the water and sediment using the enrichment factor (EF), geo-accumulation index (I_geo_), Ecological Risk Index (E_r_), and Potential ecological risk index (RI). Results showed that the concentrations of the heavy metals Pb, Cu, Cd, Al, and Fe in the water samples exceeded thresholds. Based on EF, I_geo_, and E_r_ assessment, the level of contamination by the heavy metals Pb, Cu, Cr, and Cd was found to be low, and that by Fe and Al was found to be moderate to high. The mean values of heavy metals in sediment in the descending order are as follows Fe > Al > Pb > Cu > Cr > Cd (1,445, 2692.42, 0.17, 0.048, 0.016, 0 mg/kg) respectively. Meanwhile, the mean values of heavy metals in the water in descending were Al (1.208), Fe (0.857), Pb (0.155), Cu (0.018), Cr (0.009), and Cd (0 mg/L) respectively. The sources pollution of Cu, Cd, and Pb were identified as anthropogenic sources such as city effluent, road, fisheries, and mechanic workshops. Fe and Al from sediment exhibit strong correlation (r = 0.688). This suggests that Fe and Al possibly comes from same sources originating from earth materials. In general, the potential risk assessment showed that in the Winongo River, the midstream area had higher pollution levels than the downstream and upstream areas (pollution in midstream > downstream > upstream). The sources of pollution in the midstream were identified as city effluent, roads, fisheries, and mechanic workshops. For this reason, the findings of this research are expected to provide a scientific basis for pollution control.

## Highlights

• Spatial distribution showed that the most polluted area are in the midstream areas (city).
• Heavy metals (HM) in the water were mostly exceeding the threshold guidelines.
• High amount of Fe and Al metals were found to be influenced by parent materials in the watershed.
• The EF, I_geo_, and Er generally imply that the HM contamination from anthropogenic sources is low.

## 1 Introduction

Sediments are relevant indicators of environmental pollution in the form of metal contamination, as they serve as retaining pools [1]. As surface-water resources for different purposes, such as tourism, fisheries, agriculture, industry, and being receptacles for domestic effluent, rivers can act as reservoirs of heavy metals. Heavy metals can be delivered into rivers via runoff or effluents. They can also enter aquatic ecosystems via natural processes, including geological processes such as rock weathering and meteorological processes [2]. In such cases, heavy metals that flow into reservoirs usually accumulate in the sediment and remain there for a longer time due to a lack of hydrodynamic exposure to transport them to another area [3]. For rivers, the hydrodynamic process may transport sediment to other places, or it can accumulate in the river bed sediment.

Rapid industrialization and urban development have led to large amounts of heavy metals being introduced into rivers, reducing water quality and negatively affecting the ecosystem [4]. Heavy metal accumulation also poses a threat to human health due to its biotoxicity and persistence in nature [5]. Consuming large amounts of heavy metals from water and other media can result in toxicity in humans [[6], [7], [8]]. Chronic exposure to these metals can result in cancer, anemia, and diabetes [9]. In addition, heavy metal accumulation can exacerbate ecological toxicity and influence the physical, chemical, and nutrient properties of water [10].

The Winongo River in Yogyakarta, Indonesia is critically threatened by land use changes and rapid urbanization. The watershed is divided into three zones. The upper stream area mostly consists of agricultural land and a suburban town, the middle stream area contains a city, and the downstream area consists mostly of agricultural land and a suburban town. High-intensity human activities, particularly domestic and industrial activities [11], along the Winongo River frequently and intensely flush grey water directly into the river, typically without a wastewater treatment plant (WWTP). The untreated effluent in the river, particularly in the form of heavy metals, has attracted much attention [12] because the river is also used for agricultural and fishing purposes, as well as for daily activities of most people in Yogyakarta province.

The grey water from laundry activities contains calcium (Ca), iron (Fe), cadmium (Cd), and chromium (Cr) [13]. Lead (Pb), Cr, and Cd are the most common heavy metals that are toxic to human health and can be poisonous even in small quantities [6]. These metals can easily be found in metal industries, mechanic workshops, and car emissions [14,15]. Fe, Pb, Cd, and Cr are commonly found in cultivated soil that has been treated with fertilizer containing nitrogen (N), phosphorus (P), and potassium (K) [16]. Fe and aluminum (Al) are typically found in bedrock, as the main material is volcaniclastic sediment from young Merapi deposits [17]. Six metals—Al, Fe, Pb, Cr, Cd, and copper (Cu)—were correlated to the heavy metals from each land use type in the Winongo watershed.

Heavy metals from soils and other surface areas can easily be transported by runoff and diluted in the water, making it important to evaluate the ecological risk [18,19]. Such evaluation allows for a comprehensive understanding of the heavy metal status in river water and sediment for monitoring purposes [20]. However, there is very limited information about the concentrations and spatial distribution of heavy metals and limited related research in terms of ecological risk assessment of the Winongo River. Therefore, this study aims to (1) identify the possible sources of heavy metals in the Winongo River and to reveal the spatial distribution and values of heavy metals that are closely associated with the main land uses along the river, i.e. agriculture, fisheries, local industries, metal manufacturing, car and motorbike workshop are identified. (2) To calculate the enrichment factor (EF), geo-accumulation index (I_geo_), Ecological Risk Index (E_r_), and Potential ecological risk index for Pb, Cd, Cr, Fe, Al, and Cu to evaluate the heavy metals exposure in the Winongo river.

## 2 Material and methods

### 2.1 Study area and sampling sites

**Fig. 1:**
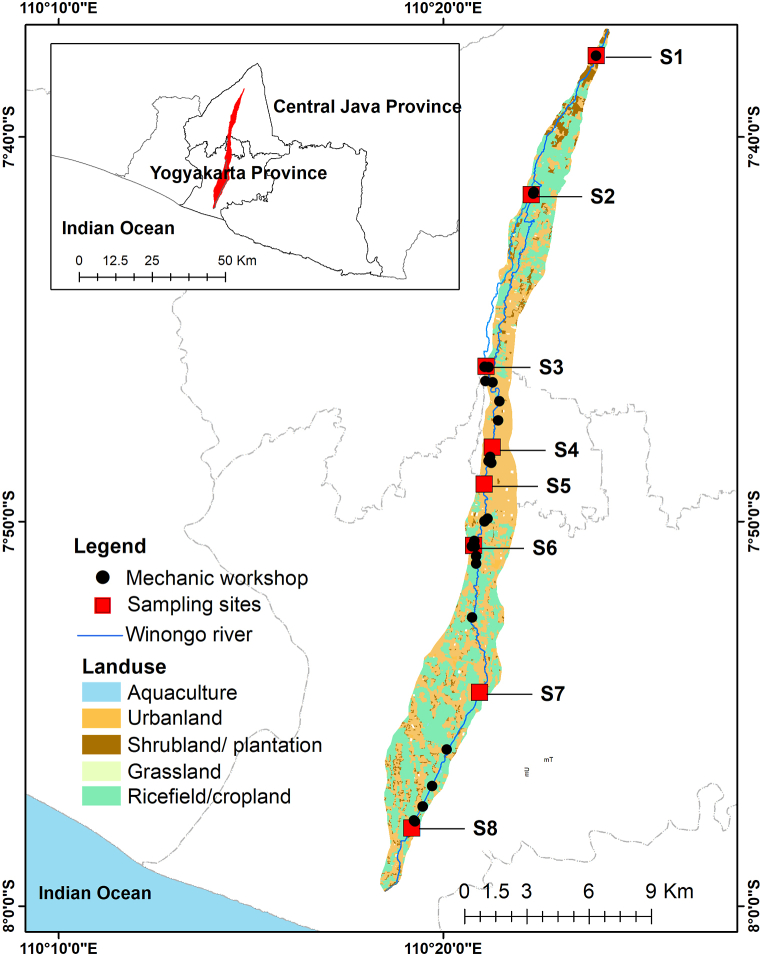
Location of the study area and sampling sites. Sampling site shown in red point and potential source of heavy metals shown by land use type and mechanic workshop distribution. The land use type was digitized from google Earth in the year of 2022. (For interpretation of the references to colour in this figure legend, the reader is referred to the Web version of this article.)

This research was conducted in the Winongo river, located in the Yogyakarta Province, Indonesia (Fig. 1). The river flows across 3 regencies among others are Sleman Regency, Yogyakarta City, and Bantul regency. The total length of the river is approximately 48 km, originating from several springs in Sleman regency. The study area lies between coordinates 7°36′49.40″ and 110°24′13.55″ to 7°59′21.75″ and 110°18′48.93”. The climate is typically a tropical climate region with the average rainfall and average temperature in 1990–2019 of 2283.6 mm and 26,5 °C respectively [21]. The Winongo watershed is situated in the young Merapi deposits, described as quaternary fluvial volcaniclastic sediment which consist mainly of sand, silt, clay and gravels [17].

In this study, water and surface sediment samples were collected in eight sampling locations in the Winongo river. The locations were selected based on river morphology, land use, local industries which produce heavy metals such as motorbike mechanic shops and metals workshops, and places related to intensive human activities that influence heavy metals in the river to provide representative heavy metal concentrations in different land use.

### 2.2 Sample collection, analysis, and quality control

Sampling procedures were performed in May 2021 and May 2022 to assess the impact of heavy metals monitoring over the course of a year. Eight samples of sediment and eight samples of river water were collected during the rainy season in May of 2021, and eight samples of sediment and eight samples of river water were collected during the rainy season in May of 2022. In total, 16 samples of sediment and 16 samples of river water were collected in different land use areas for analysis [18]. Sediment samples were collected at a depth of 0 cm–5 cm using a grab sampler, stored in plastic bags, and preserved in an icebox (−4 °C) [2]. Meanwhile, the water samples were transferred to bottles, acidified using HNO_3_, and kept in an icebox [22]. The sediment and water samples were sent to a laboratory for analysis within 24 h [23,24]. The water samples were filtered and then analyzed using an atomic absorption spectrophotometer (AAS) to determine Pb, Cd, Cr, Fe, Al, and Cu concentrations.

All the sediment samples were naturally dried in a laboratory for 3 weeks and then sieved through a 1.0 mm mesh nylon sieve. Next, 0.3 g of dried samples were wetted with 25 ml HNO_3_ (65%) and heated at 95 °C for 10 min. After cooling at room temperature (25 °C), 10 ml of HNO_3_ was added to the samples. Later, the digested samples were filtered and diluted in 50 ml of distilled water. Finally, the samples were analyzed to determine the Pb, Cd, Cr, Fe, Al, and Cu content using an AAS. Quality control measures were taken to improve the accuracy of the analysis, including the preparation of blank samples from stock solution (1000 ppm) and the use of a calibration curve. Nitric acid (HNO^3^) was purchased from a certified supplier (Merk EMSURE reg PH Eur, ISO) with the registration number 01-2119487297-23-XXXX. The data analysis was performed using standard laboratory operating procedures according to ISO 17025:2017.

### 2.3 Heavy metal pollution assessment in water and sediment

#### 2.3.1 Heavy metal assessment in the water

The heavy metal concentration from the surface water samples were analyzed descriptively by comparing the water quality from the measurement with the WHO guideline value for heavy metals [25] and Indonesia Water Quality Standard (WQS) on Government regulation No. 22 of 2021 of Class I and Class II. The Indonesia water quality standard Class I regulated the drinking water or domestic use guideline, meanwhile for the class II were used to regulate water quality for recreation, aquaculture, and agriculture [26].

#### 2.3.2 Heavy metal assessment in sediment

##### 2.3.2.1 Enrichment factor (EF)

Enrichment factors (EF) were used to determine the activities and anthropogenic impact on heavy metal concentration in the sediment [27]. In EF calculation, background samples were important to understand the element abundance in the environment by comparing the metal concentration with the other concentrations from the background samples. Because there was limited information about the background concentration of heavy metals in the Winongo River, based on recent research the background samples for Pb, Cu, Cd, Cr, Fe, and Al are 9.01, 14.1, 1.24, 1.56, 1501.22, 15,992.7 mg/kg respectively [28]. These background samples are also used for I_geo_ calculation is shown in Equation (1)

The enrichment factor is expressed as follows [27,29]:

(1) $$EF=\frac{\left(\frac{Ci}{Cref}\right)sampel}{\left(\frac{Ci}{Cref}\right)background}$$

where C is the concentration of element “i” in the sediment samples, C is the concentration of reference elements in sediment samples, EF is the ratio of the concentration of element being tested from the sample and element from the background. Enrichment factors were classified into five levels as listed in Table 1.

**Table 1:** Enrichment factor classification.

Enrichment factor	Classification
1 < EF < 2	Minimum enrichment
2 < EF < 5	Medium enrichment
5 < EF < 20	Adequate enrichment
20 < EF < 40	High enrichment
EF > 40	Very high enrichment

### 2.4 Geo-accumulation Index (Igeo)

The geo-accumulation index is one of the pollution index parameters to determine the toxic level of sediment by comparing the current concentration of heavy metals with the background level can be calculated using Equation (2) [30,31]:

(2) $$Igeo=\mathrm{Log}\;2\frac{\left(Cn\right)}{1.5\;\left(Bn\right)}$$

where C_n_ is the concentration of element “n” in sediment samples and Bn is the reference level of heavy metal “n” in nature. Factor 1.5 was applied in the equation as a background correction due to lithogenic variation. It also represents a small anthropogenic influence on the sediments [32]. The geoaccumulation index is divided into 7 classes from unpolluted to extremely polluted as shown in Table 2 [31].

**Table 2:** Classification of pollution level based on Igeo.

Geoaccumulation index Igeo	classification
Igeo ≤ 0	Unpolluted
0 < Igeo <1	Unpolluted to moderately polluted
1 < Igeo <2	Moderately polluted
2 < Igeo <3	Moderately polluted to heavily polluted
3 < Igeo <4	Heavily polluted
4 < Igeo <5	Heavily polluted to extreme polluted
Igeo >5	Extreme polluted

### 2.5 Potential ecological risk assessment (E_r_)

The potential ecological risk (E_r_) is used to assess overall contamination by combining ecological risk elements, such as contamination factors (C_f_) and toxic factors of an individual element (T_r_) during pre-industrial [29]. The formula is written in Equations (3), (4), (5)) as follows

(3) $$E_{r}^{i}=T_{r}^{i}C_{f}^{i}$$

(4) $$C_{f}^{i}=\frac{C_{n}^{i}}{C_{o}^{i}}$$

(5) $$RI=\Sigma E_{r}^{i}$$

where E^i^_r_ is the ecological risk factor for heavy metal. T^i^_r_ is the toxic-response factor for a metal, which is defined for Cu=Pb=Al = 5, Cr = 2, Fe = 6, and Cd = 30 [29,32]. C^i^_f_ is the contamination factor, C^i^_n_ is the reference value for metal, C^i^_o_ is the concentration of metal in sediment, and RI is the total of all risk factors for heavy metals. Five risk levels were classified based on the potential ecological risk index as shown in Table 3.

**Table 3:** Potential ecological risk index is classified into 4 classes [33].

Er Value	Potential ecological risk index	Classification
Er < 40	<150	Low ecological risk or low ecological pollution level
40 <Er < 80	150 < RI < 300	Moderate ecological risk or moderate ecological pollution level
80 <Er < 160	300 < RI < 600	Considerable ecological risk or severe ecological pollution level;
160 <Er < 320	RI > 600	High ecological risk or serious ecological pollution level
Er > 320	–	Very high risk

### 2.6 Data analysis

The statistical analysis conducted with Origin 2022 to compare the relationship between samples. By considering the uncertainty in source identification, the principal component analysis used to identify the possible sources of heavy metals in the Winongo river to minimize the uncertainties during descriptive analysis [34,35]. Pearson matrix analysis was performed to indicate the level of correlation between heavy metal in the water and sediment. The p value 0.05 indicates strong and significant correlations [7]. The spatial distribution of heavy metals in the Winongo river sediment, the inverse distance weighted (IDW) interpolation were used to show the distribution of Cd, Cr, Cu, Pb, Al, and Fe in the river and the area of interpolation. With an assumption it can represent the nearest sampling point value. It is used to identify the possible contamination [36]. The buffer zone of the river is also determined to recognize pollution sources near the sampling sites.

## 3 Results and discussion

### 3.1 Heavy metal analysis in the surface water

Average heavy metal concentrations in the surface water samples from two different time samplings results are listed in Table 4. The concentration of Pb, Cu, Cr, Cd, Fe and Al of Winongo river varied between 0.1 and 0.69, 0–0.04, 0–0.02, 0–0.01, 0.2–1.68, and 0.47–3.31 mg/L. According to the Special Region of Yogyakarta Governor Regulation Number 22 of 2007 concerning water class regulation in the Special Region of Yogyakarta, Winongo Rivers refers to Water Quality Standard (WQS) of class one (I) which can be used for drinking water. However, the Indonesian Government usually use the WQS based on the given class for each river. For that reason, the use of WHO standards in this research is to compare with the other river in the world.

**Table 4:** Average heavy metal concentration in water sample of Winongo river from different land use.

Sites	Watershed	Coordinate	land use	Pollution Sources	Pb (mg/L)	Cu (mg/L)	Cr (mg/L)	Cd (mg/L)	Fe (mg/L)	Al (mg/L)
1	Upstream	−7.631510; 110.399949	Suburban town	Mechanical workshop, domestic	0.14	0.04	0.01	0.01	0.59	0.71
2		−7.691878; 110.371659	Suburban town	Agriculture, domestic waste disposal	0.26	0.03	0.01	0.00	0.50	1.45
3	Midstream	−7.766185; 110.352029	Suburban town	Mechanical workshop, domestic, road	0.02	0.00	0.02	0.00	1.68	2.14
4		−7.801229; 110.354797	City	Domestic, road	0.69	0.04	0.01	0.00	1.54	3.31
5		−7.817345; 110.351273	City	Fisheries, domestic	0.04	0.03	0.01	0.00	1.37	0.88
6		−7.843820; 110.346668	Suburban town	Welding workshop, domestic	0.10	0.03	0.01	0.00	1.19	0.47
7	Downstream	−7.907590; 110.349300	Suburban town	Agriculture, domestic waste, duck farmland	0.43	0.02	0.01	0.00	0.20	2.47
8		−7.966403; 110.319848	Agriculture area	Agriculture, bike repair shop, road	0.40	0.00	0.00	0.00	1.18	0.62
	Guideline value for drinking water^a^				0.01	0.05	2	0.003	–	–
	Indonesian WQS Class I				0.03	0.02	0.05	0.01	0.3	–
	Indonesian WQS Class II				0.03	0.02	0.05	0.01	–	–

a WHO Guideline for drinking water (2022).

**Fig. 2:**
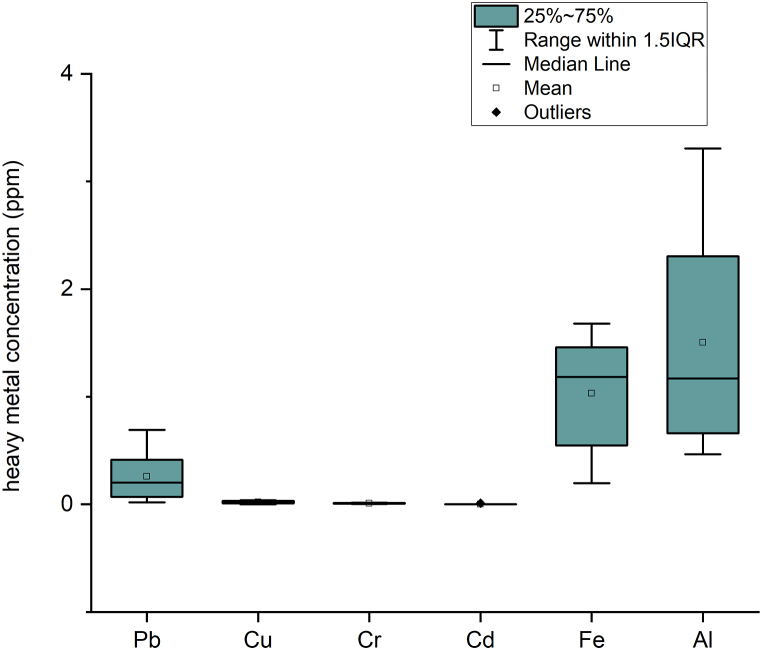
Heavy metal concentration in the surface water.

Table 4 shows only Pb and Cd significantly exceed the WHO maximum concentration limit for drinking water of 0.01 and 0.003 mg/L, respectively. However, based on Indonesia WQS the heavy metal concentration in Winongo river mostly exceeds the Indonesia WQS of Class I for Cd, Cu, Cd and Fe. Overall, regarding this result, water quality concentration for heavy metals in the Winongo river is higher than the threshold value of the Indonesia WQS of Class II and WHO guidelines. It is also shown that the Wingono river needs further research for agriculture, aquaculture, and tourism water uses. Compared with other rivers in Indonesia, such as Damsari River, Jabawi River, Kleblow River, and Komba river [26], the Pb,Cd, Cu, and Fe in Winongo river were higher, compared to the others river in the world the concentration of Pb, Cu, Cr and Cd were lower than Bhairab River [5], Shanghai River [37] and Fe, Pb and Cu also higher than Belik River in Yogyakarta [38]. The distribution of means for the measured element in descending order was Al > Fe > Pb > Cu > Cr > Cd (Fig. 2).

The average concentration of lead (Pb) in the Winongo River was varied, ranging from 0.02 to 0.69 mg/L. Notably, all the samples were exceeding the standard for WHO guidelines. It was indicated that the influence of metal pr mechanic workshop near the sampling sites has significant impact for the increasing of Pb value in the water (Table 4). Lead can be found as a pollutant in several activities, for example the lead from gasoline of motorized vehicles presence as media as well as tires will transfer the residues which contain lead material to soil and water. It was found that Site S1, S2, S4, S6, and S8 which was next to the mechanic workshop have higher value of Pb (Fig. 1).

Compared to the WHO standard (0.05 mg/L), the average Cu concentration in all collected samples was below the guideline limits. However, compared to the Indonesian WQS (0.02 mg/L), it was above the limits. There are five sites whose values exceed the WQS (Sites S1, S2, S4, S5, and S6). S4 and S5 are located in the middle of a densely populated city. Cu can come from effluent of agricultural activities and livestock manure. However, domestic activities, traffic, and mechanical workshops seem to contribute to higher Cu emissions. This shows that the spatial distribution of Cu concentration in the surface sediment is higher in urban and suburban areas with many mechanical workshops (Fig. 1). The excess of heavy metal Cu is dangerous because it can cause mucosal irritation, central nervous system irritation, hepatic and renal damage [39].

On the other hand, the concentration of Cr in the Winongo River is still below the quality standard of WHO (2 mg/L) and Indonesia WQS of Class I and Class II (0.05 mg/L). Cr is a heavy metal that cannot be decomposed, and it still needs to be monitored since it comes from motor vehicles and fertilizer [2]. Cr metal eaten by biota will settle in the body and is certainly dangerous if consumed by humans. The concentration of Cd in some sampling points was below the detection limit except for S1. Cadmium was only found at S1 with a concentration of 0.01 mg/L. S1, which is located in a suburban town and associated with mechanic workshops and settlements, is the source of Cd pollution. Metal workshops, metal industry, and coal combustion are considered to be the main source of Cd [7]. In the natural stages, the main cadmium sources are from atmospheric decomposition and the earth's crust. Cd is the most transportable heavy metal and it is easily transported from soil to the water and other media [40].

In addition, all the collected samples had Fe and Al concentrations above the guideline values. Despite that, Fe and Al are not significantly harmful to human bodies and aquatic biota. The average concentration of Fe is in the range of the mean value of 1 mg/L. The highest Fe concentration was found at site 3, which is a battle between two tributaries. The source of Fe pollution in site 3 is possibly coming from the mechanic workshop and the runoff from the road, which is located close to a bridge that has heavy traffic. The Fe content in river water can also come from nature and human activities. Meanwhile, Al shows a concentration that tends to be large compared to the concentration of other heavy metals, ranging from 0.47 to 3.31 mg/L. The guideline value of Al for drinking water based on WHO guidelines [25] does not exist as it is usually used as a coagulant in water treatment.

### 3.2 Sediment quality analysis

#### 3.2.1 Spatial distribution of heavy metal in Winongo river sediment

**Fig. 3:**
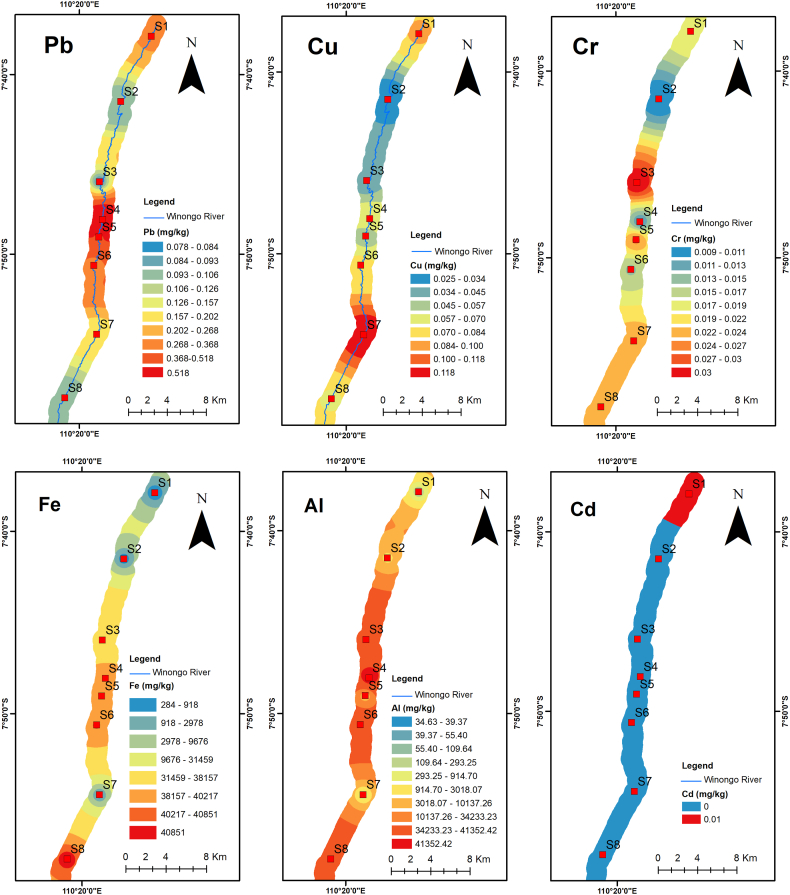
The spatial distribution of average heavy metal pollution in the sediment samples map of Winongo River.

The range of metals in the sediment was 0.074–0.518 (mg/kg) for Pb, 0.017–0.119 (mg/kg) for Cu, 0.008–0.03 (mg/kg) for Cr, 11.82–40,851.1 (mg/kg) for Fe, 33.24–41,352.43 (mg/kg) for Al, and 0–0.01 (mg/kg) for Cd. The results presented in Fig. 3 and Table 5 revealed that the midstream had the highest value of heavy metals in the sediment, followed by the downstream area and upstream area. Hence, the midstream area has a high pollution level as it is associated with rapid urbanization, industrial effluent, and mechanic workshops effluent.

**Table 5:** Average heavy metal concentration in sediments of Winongo river from different land use.

Sites	Watershed	Coordinate	land use	Pollution Sources	Pb (mg/kg)	Cu (mg/kg)	Cr (mg/kg)	Cd (mg/kg)	Fe (mg/kg)	Al (mg/kg)
1	Upstream	−7.631510; 110.399949	Suburban town	mechanical workshop, domestic	0.21	0.07	0.02	0.01	22.86	33.24
2		−7.691878; 110.371659	Suburban town	agriculture, domestic waste disposal	0.08	0.02	0.01	0	9.27	9.08
3	Midstream	−7.766185; 110.352029	Suburban town	mechanical workshop, domestic, road	0.07	0.03	0.03	0	32.83	39.89
4		−7.801229; 110.354797	City	Domestic, road	0.52	0.06	0.01	0	48.97	56.26
5		−7.817345; 110.351273	City	Fisheries, domestic	0.36	0.04	0.02	0	42.10	39.78
6		−7.843820; 110.346668	Suburban town	Welding workshop, domestic	0.25	0.06	0.01	0	54.15	35.99
7	Downstream	−7.907590; 110.349300	Suburban town	Agriculture, domestic waste, duck farmland	0.14	0.12	0.02	0	11.82	36.96
8		−7.966403; 110.319848	Agriculture area	Agriculture, bike repair shop, road	0.09	0.06	0.02	0	51.94	37.64
				Mean	0.17	0.05	0.02	0.01	28.82	32.83

**Fig. 4:**
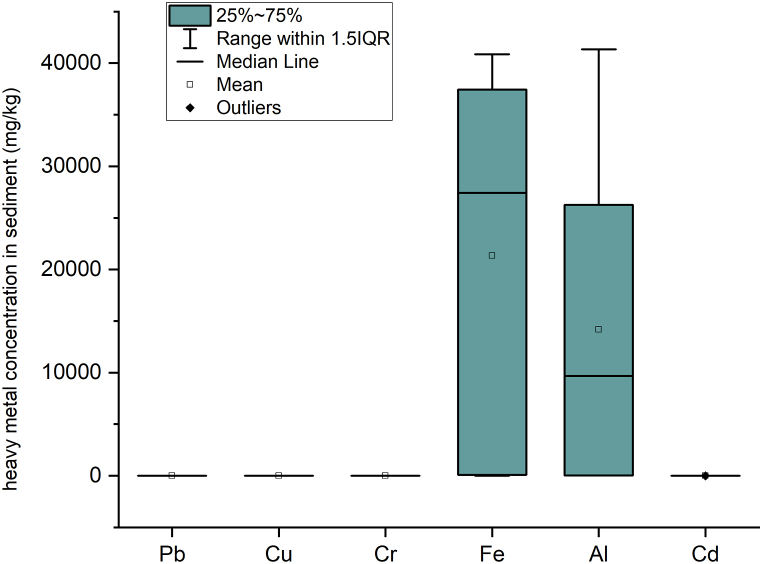
Heavy metal concentration in the river sediment. The heavy metal concentrations in the river sediment of Winongo River show that iron (Fe) and aluminum (Al) have the largest range of concentrations, as their concentrations are very high compared to other elements.

Heavy metal concentrations in the river sediment and the considered pollution sources are shown in Table 5. The mean concentration for heavy metals in ascending order is as follows: Cd < Cr < Cu < Pb < Al < Fe (Fig. 4). The Fe concentration was the highest concentration among all the elements in the river sediment. This might have happened due to the fact that the lithogenic material in the Winongo river is a young volcanic deposit which contains [17,41]. The mean concentration of Fe was found to be 2692.42 mg/kg. Eventually, the findings for this study are higher than the background value of 1501.22 mg/kg [28] and the world average value of 47,200 mg/kg [42]. The concentration of Al was considered not harmful in the sediment.

Among the six elements, the mean concentration of Pb is the third highest at 0.17 mg/kg. This value is generally lower than the background value of 9.01 mg/kg [28], as well as the world average value of 20 mg/kg [42]. The possible sources of Pb in the study area include gasoline residue, gasoline additives, metal extraction, and highways that use asphalt (Table 5). The mean values of Cu (0.048 mg/kg), Cr (0.016 mg/kg), and Cd (0 mg/kg) were also observed to be particularly lower than other heavy metals. Consequently, they are also lower than the background value and average world concentration. The average world concentration for Cu, Cr, and Cd are 45, 90, and 0.3 mg/kg respectively [42].

#### 3.2.2 Pollution assessment in river sediment

**Fig. 5:**
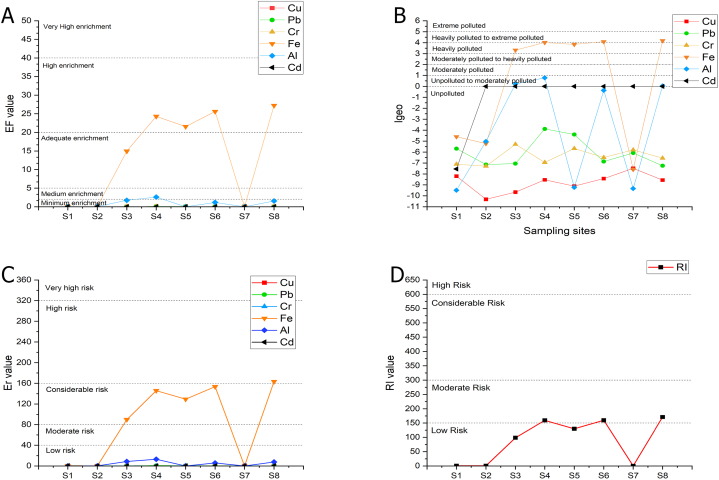
(A) Enrichment factor (EF), (B) Index geoaccumulation (Igeo), (C)Ecological risk value (E_r_), and (D) Potential ecological risk Index (RI) of heavy metals results in Winongo River, Indonesia.

To measure the pollution level in the river sediment, all sampling sites were evaluated using Enrichment factor (EF), geoaccumulation index (I_geo_), Ecological risk index (Er), and potential ecological risk index (PEI). The results of EF and I_geo_ are shown in Table 6, while the results for Er and PEI are presented in Table 7. From Fig. 5, it was revealed that the spatial distribution of heavy metals has significantly higher pollution, particularly for Fe metal, in the midstream at sampling sites S3, S4, S5, and S6, and also in the downstream area in site S8. The main sources of pollution in the midstream, as listed in Table 5, are domestic effluent, road runoff, mechanic workshop effluent, fishpond, and dunk farmland. This is typical anthropogenic pollution in the urban area [4]. However, in the downstream area, the region with the highest degree of pollution was site S8. The reason was that S8 is close to agriculture, bike repair shops, roads, and the accumulation of pollution as well.

**Table 6:** Enrichment factor assessment of heavy metals in Winongo River.

Sites	EF						Igeo					
	Cu	Pb	Cr	Fe	Al	Cd	Cu	Pb	Cr	Fe	Al	Cd
S1	0.005	0.029	0.011	0.062	0.002	0.008	−8.20	−5.68	−7.10	−4.60	−9.50	−7.54
S2	0.001	0.011	0.010	0.040	0.046	0.000	−10.32	−7.14	−7.29	−5.22	−5.03	–
S3	0.002	0.011	0.038	14.970	1.741	0.000	−9.67	−7.05	−5.29	3.32	0.22	–
S4	0.004	0.102	0.012	24.289	2.586	0.000	−8.54	−3.88	−6.94	4.02	0.79	–
S5	0.003	0.071	0.029	21.564	0.002	0.000	−9.12	−4.39	−5.67	3.85	−9.24	–
S6	0.004	0.013	0.017	25.590	1.165	0.000	−8.43	−6.86	−6.49	4.09	−0.36	–
S7	0.008	0.022	0.027	0.008	0.002	0.000	−7.48	−6.08	−5.80	−7.57	−9.34	–
S8	0.004	0.010	0.016	27.212	1.542	0.000	−8.56	−7.25	−6.55	4.18	0.04	–

**Table 7:** Potential Ecological Risk Assessment of heavy metals in Winongo River.

Sites	Er						RI
	Cu	Pb	Cr	Fe	Al	Cd	
S1	0.03	0.15	0.02	0.37	0.01	0.24	0.82
S2	0.01	0.05	0.02	0.24	0.23	0.00	0.55
S3	0.02	0.06	0.08	89.82	8.71	0.00	98.68
S4	0.04	0.51	0.02	145.73	12.93	0.00	159.23
S5	0.03	0.36	0.06	129.38	0.01	0.00	129.84
S6	0.04	0.06	0.03	153.54	5.83	0.00	159.51
S7	0.08	0.11	0.05	0.05	0.01	0.00	0.31
S8	0.04	0.05	0.03	163.27	7.71	0.00	171.10

Enrichment Factor (EF) for each heavy metal in the Winongo river is shown in Table 6. Overall, the mean EF for heavy metals was ordered as Fe > Al > Pb > Cr > Cu > Cd. From the spatial distribution on EF in Fig. 5, the EF values indicate low to moderate pollution from anthropogenic activities. In a total of 48 data (6 metal parameters on 8 sampling sites), the remaining 8.33% were classified as high enrichment, 2.08% as adequate enrichment, and 89.58% as minimum enrichment. The EF of Fe and Al is considered higher than other metals; however, the understanding of Fe and Al as a major element in the earth's crust may come from weathering processes from the lithologic material in the surrounding river [22]. It is suggested that Fe and Al are not significant metals for sediment pollution. In contrast, the other elements of metal that have the EF value < 1.5 are suggested by anthropogenic sources even though they have low enrichment.

Based on the I_geo_ calculation, the majority of sampling stations experienced unpolluted contamination. The pollution levels were mainly unpolluted (83.33%), unpolluted to moderately polluted (6.25%), heavily polluted (6,25%) and heavily polluted to extremely polluted (4.17%). The I_geo_ of Fe and Al was significantly higher than other elements, whereas for other heavy metals, they were practically uncontaminated (I_geo_ ≤ 0) [5] with Pb, Cu, Cd, and Cr. The pollution level of Fe was considered heavily polluted in sites S3, S4, and S5 and heavily polluted to extremely polluted in sites S6 and S8. Besides, for all pollution levels found to be unpolluted to moderately polluted in Site S3, S4, and S8.

The Ecological risk index (E_r_) shows the risk index in the river sediment. From Table 7 and Fig. 5, the pollution level in the Winongo river varies from low risk to high risk. From the samples, 87.5% were identified as low risk, particularly for Pb, Cu, Cr, and Cd (Er < 40). The 10.43% were classified as considerable risk (80 ≤ Er ≤ 160), and the remaining 2.08% were deemed high risk (160 ≤ Er ≤ 320). High-risk levels were only found in site S8 for Fe metals. No moderate or very high-risk level of pollution was detected at the sampling sites. To some extent, the Er value for all sampling sites indicates a high potential ecological risk for Fe, while for other metals, the potential ecological risk may be expressed as low pollution [37].

**Fig. 6:**
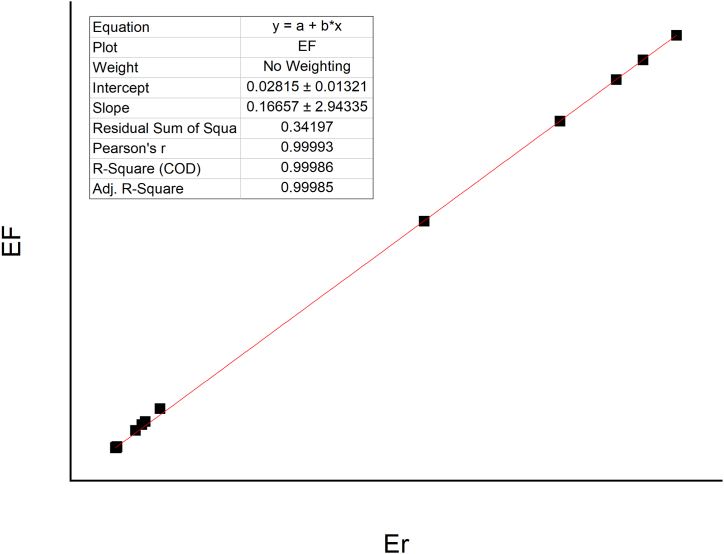
Correlation of enrichment factor and ecological risk value shows good comparison.

Fig. 6 displays a strong correlation between Er and FE values. In accordance with the Er, RI values show that 62.5% indicate low risk (RI < 150) and 37.5% are categorized as moderate risk (150 = RI ≤ 300). The spatial distributions of risk from RI values ranking from highest to lowest are S8>S6>S4>S5>S3>S1>S2>S7. The highest RI values were found at the site S8, located downstream near an agricultural area and mechanic workshops. The lowest RI values were found at the site S7, which is correlated with agriculture, farm, and domestic waste sources (see Fig. 1). The values of individual metals in the sediment indicate that Fe and Cd pose lower risks compared to the other sampling sites. This is because the RI is calculated as the sum of metals units [5]. Moreover, the S7 sampling site is not exposed to metal workshops, and the Fe in the sediment is likely linked to the effluent discharge from metal activities [7]. Overall, the midstream RI and Er values were higher than those in the downstream and upstream areas (RI midstream > RI downstream > RI upstream), reflecting the strongest anthropogenic impact from the city, followed by suburban towns and agricultural areas.

#### 3.2.3 Source identification of heavy metal in the river sediment

**Fig. 7:**
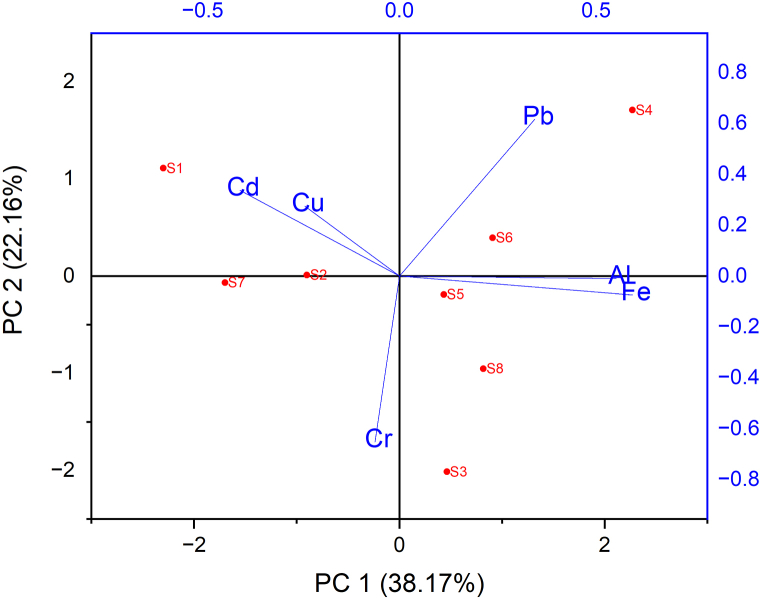
Principal component analysis of the river sediment (n = 48).

The results of a principal component analysis (Fig. 7) show that PC1 (38.17%) and PC2 (22.16%) contributed to the total variance and together explained 60.33% of the total variance. This corresponds to the complex source of pollution in the midstream, which comes from both non-point source runoff and point source effluent. A Pearson correlation matrix for the sediment sample (Table 8) suggests that Fe and Al have a strong correlation (r = 0.688), and their loading in PC1 indicates that they originate from the same sources, such as natural sources like earth crust material. Fe and Al also show a good positive correlation in both the water and sediment samples (r = 0.795) (Table 9).

**Table 8:** Pearson correlation matrix analysis for heavy metals in the sediment of Winongo river.

	Pb	Cu	Cr	Fe	Al	Cd
Pb	1					
Cu	0.065	1				
Cr	−0.345	0.060	1			
Fe	0.423	−0.234	0.09	1		
Al	0.329	−0.190	−0.025	0.688	1	
Cd	−0.014	0.209	−0.037	−0.465	−0.352	1

**Table 9:** Pearson correlation matrix analysis between heavy metals in the water and sediment of Winongo river.

	Pb	Cu	Cr	Fe	Al	Cd
Pb	1					
Cu	0.0002	1				
Cr	−0.351	0.364	1			
Fe	0.099	0.259	0.430	1		
Al	0.088	0.209	0.307	0.795	1	
Cd	−0.038	0.300	0.125	−0.168	−0.142	1

Although Cd, Cu, and Pb are typically associated with anthropogenic impacts [34], elements from PC2 suggest that Pb has high loads and may come from specific inputs, such as mechanic workshops and runoff from asphalt roads. However, Cd and Cu are thought to come from urban waste disposal [43]. In sediment samples, Cu shows a positive correlation with Cd (r = 0.209), and in previous studies, Cr elements were indicated to be associated with the parent material [35]. Pb also exhibits a positive correlation with Al (r = 0.329) and Fe (r = 0.432). Overall, the correlation matrices between heavy metals in the sediment and water demonstrate a positive relationship for several elements. For example, Cu shows a positive correlation with Cr (r = 0.364), Fe (r = 0.259), Al (r = 0.209), and Cd (r = 0.300). Cr in the water also shows a positive correlation with Fe (r = 0.430), Al (r = 0.307), and Cd (r = 0.125). This suggests that heavy metals may come from other environmental factors, such as soil and waste on the riverbank.

Even though the low loading of Cr indicates a small enrichment of the metal, results for some heavy metals (Pb, Cu, Cr, and Cd) suggest low-risk sediment. However, heavy metals in the water and sediment should still be continuously monitored in the Winongo River because it is used for agriculture, domestic purposes, and fisheries. Moreover, land use changes in the future may lead to an increase in heavy metals in the river water and sediment. Hence, to manage the pollution from agriculture, artificial fertilizer should be reduced [44] and WWTPs should be installed for domestic industries.

## 4 Conclusion

In this study, heavy metal pollution in the water and sediment of the Winongo River was analyzed using spatial distribution, the enrichment factor (EF), the geoaccumulation index (I_geo_), the ecological risk index (Er), and the potential risk index (RI). The results showed that heavy metals (Pb, Cu, Cd, Al, and Fe) in the river water exceeded threshold values according to WHO and Indonesian class II water quality standards. The mean concentration of the measured elements in the water were Al > Fe > Pb > Cu > Cr > Cd, with mean values in descending order of 1.208 mg/L, 0.857 mg/L, 0.155 mg/L, 0.018 mg/L, 0.009 mg/L, and 0 mg/L, respectively. The spatial distribution of heavy metals in the water indicates that the concentrations in the midstream are higher than in the other areas, with the pollution coming from the city, roads, mechanic workshops, and fisheries.

However, the results of heavy metal analysis and pollution assessment using EF, I_geo_, and Er generally imply that the Pb, Cu, Cr, and Cd contamination from anthropogenic sources is low. The mean values of heavy metals in sediment in descending order were Cd < Cr < Cu < Pb < Al < Fe (1445 mg/kg, 2692.42 mg/kg, 0.17 mg/kg, 0.048 mg/kg, 0.016 mg/kg, and 0 mg/kg), respectively. In contrast, Fe and Al show higher values and a higher pollution index than other elements according to EF, I_geo_, and Er assessments, suggesting that the pollution source comes from the lithological impact in the Winongo River. The RI and the Er results indicate a low to moderate risk, with the moderate risk distributed in the midstream area (city) and the downstream area (suburban and agricultural area). Overall, the spatial distribution of heavy metals indicates that pollution levels are highest in the midstream area, followed by the downstream and upstream areas. Based on the PCA and Pearson's correlation matrix, several elements are suggested to possibly come from similar sources, such as Fe and Al, which may come from earth's crust materials. However, Cu, Cr, Cd, Fe, and Al are also proposed to come from similar sources, such as soil from non-point sources or domestic waste along the riverbank.

Research on heavy metals risk in Indonesia, particularly in the Winongo river was limited, therefore, this study has limited information on sediment age and sediment background. However, rivers in Indonesia typically have similar pollution sources, such as agricultural, domestic, and industrial waste. Therefore, this research can serve as a basis for heavy metals assessment in developing countries, particularly in Indonesia. In the future, WWTPs for domestic industries should be regulated by the government to reduce heavy metals pollution.

## Author contribution statement

Lintang Nur Fadlillah: Conceived and designed the experiments; Analyzed and interpreted the data; Wrote the paper. Sri Utami: Contributed reagents, materials, analysis tools or data; Wrote the paper. Alfina Ayu Rachmawati: Contributed materials, analysis tools or data; Wrote the paper. Galih Dwi Jayanto: Contributed reagents, materials, analysis tools or data. M. Widyastuti: Analyzed and interpreted the data; Wrote the paper.

## Data availability statement

Data included in article/supplementary material/referenced in article.

## Funding

This work is supported by Faculty of Geography, Gadjah Mada University, Indonesia.

## Declaration of competing interest

The authors declare no conflict of interest that are relevant to this research.
